# Impact of heavy metal lead stress on polyamine levels in *Halomonas BVR* 1 isolated from an industry effluent

**DOI:** 10.1038/s41598-017-13893-0

**Published:** 2017-10-18

**Authors:** Manasi, Sridev Mohapatra, N. Rajesh, Vidya Rajesh

**Affiliations:** 1 Department of Biological Sciences, Birla Institute of Technology and Science, Pilani-Hyderabad Campus, Jawahar Nagar, Shameerpet Mandal, R.R. Dist, 500 078 India; 2 Department of Chemistry, Birla Institute of Technology and Science, Pilani-Hyderabad Campus, Jawahar Nagar, Shameerpet Mandal, R.R. Dist, 500 078 India

## Abstract

In living systems, environmental stress due to biotic and abiotic factors triggers the production of myriad metabolites as a potential mechanism for combating stress. Among these metabolites are the small polycationic aliphatic amine molecules - polyamines, which are ubiquitous in all living organisms. In this work, we demonstrate a correlation between cellular concentration of three major polyamines (putrescine, spermidine and spermine) with lead exposure on bacteria for a period of 6–24 h. We report that indigenously isolated *Halomonas* sp. strain *BVR* 1 exhibits lead induced fluctuations in their cellular polyamine concentration. This response to lead occurs within 6 h post metal treatment. During the same time interval there was a surge in the growth of bacteria along with an enhancement in the putrescine levels. We conclude that in *Halomonas sp*. strain *BVR* 1, an early response is seen with respect to modulation of polyamines as a result of lead treatment and hypothesize that endogenous polyamines contribute towards scavenging lead in these bacteria.

## Introduction

Environmental stress has a significant negative impact on the growth of bacteria. Irrespective of their habitat, bacteria are constantly exposed to a variety of environmental stresses such as salinity, heat, metal stress etc. and they have evolved numerous mechanisms to combat them^1^. Sensing and responding correctly to these stresses is crucial for the survival of all bacteria^2^. Stress in bacteria leads to increased metabolite production due to changes in physiological processes contributing to their protective mechanisms^3^. These stress related adaptive and protective responses include, alteration of gene expression patterns, changes in the control of transcription, translation, stability of transcripts and proteins etc^4^. Among the various environmental stresses affecting bacteria, metal stress is critical as metals are not degraded biologically, and their bioaccumulation tendency renders them highly toxic^5^.

Accumulation of a diverse range of metabolites involving amino acids and amines is commonly seen in response to exposure to heavy metals. This indicates that fluctuations in nitrogen metabolism is central to the heavy metal response^1,6^. Among these metabolites, the major players are polyamines (PA), which are ubiquitous polycationic aliphatic amines present in all living organisms. The three major PAs include putrescine, spermidine and spermine^7^.

Putrescine, the diamine precursor to spermidine and spermine is synthesized from amino acid arginine and ornithine via the enzymes arginine decarboxylase (ADC) and ornithine decarboxylase (ODC) respectively^8,9^.The aminopropyl moieties for the synthesis of spermidine and spermine are donated by decarboxylated S-adenosylmethionine^10^. The PA metabolic pathway is intricately connected to the metabolism of several amino acids and other important metabolites like ethylene and γ-aminobutyric acid (GABA), thus forming a crucial, complicated network of nitrogen sequestration. Though, the direct and indirect roles of PAs for metal sequestration in plants are well documented^9,11–13^, there are limited reports on their role in bacteria. Joutey *et al*.^14^ reported that inhibition of polyamine synthesis led to inhibition of chromium tolerance. Rhee *et al*.^15^ also reported the divergent roles of polyamines under various stress responses in bacteria.

The polycationic chemistry of these molecules provides direct structural evidence for their roles in metal sequestration through lone-pair-bond-pair reactions^16^. It has also been confirmed that, during heavy metal stress, plants synthesize polyamines along with molecules such as proline, histidine, glutathione, phytochelatin, γ-glutamylcysteine proving that nitrogen metabolism is central to the response to heavy metals in plants^1^. For example, treatment of oat seedlings with CdCl_2_ resulted in a 10-fold increase in the levels of putrescine^7^. An increase in plant putrescine levels followed by a concomitant increase in spermidine and spermine levels is also documented with copper^17^.

The following mechanisms have been proposed justifying the increase in PA levels and their precise role in scavenging metals: (a) Heavy metal stress leads to the production of OH $\mathrm{and}$ O_2_^−^ due to oxidative stress^18^, leading to disintegration of biomembranes by lipid peroxidation. Polyamines are known to scavenge free radicals *in-vitro*. (b) Polyamines block one major vacuolar channel, and their accumulation decreases inward ion conductance at the vacuolar membrane to facilitate metal ion compartmentation^19^. (c) Polyamines being positively charged tend to bind to metals and nucleic acids for their stability and can also help in sequestration of metals^20^.

Thus, it is clearly evident that the potential role of PA in modulating plant stress due to heavy metals is well documented^21^, but there are very few reports on PA mediated regulation of metals stress in bacteria. Hence, this work aims to identify the link between regulation of polyamine metabolism and heavy metal treatment. Our study, a first of its kind, analyzes the three centered interaction between polyamines, heavy metal and the physiology of *Halomonas sp*. strain *BVR* 1 in the presence of lead. These results pave way for further experimentation and explanation on the association of polyamines in bacteria and heavy metals.

## Results and Discussion

### Bacterial growth in the presence of Heavy Metal

**Figure 1:**
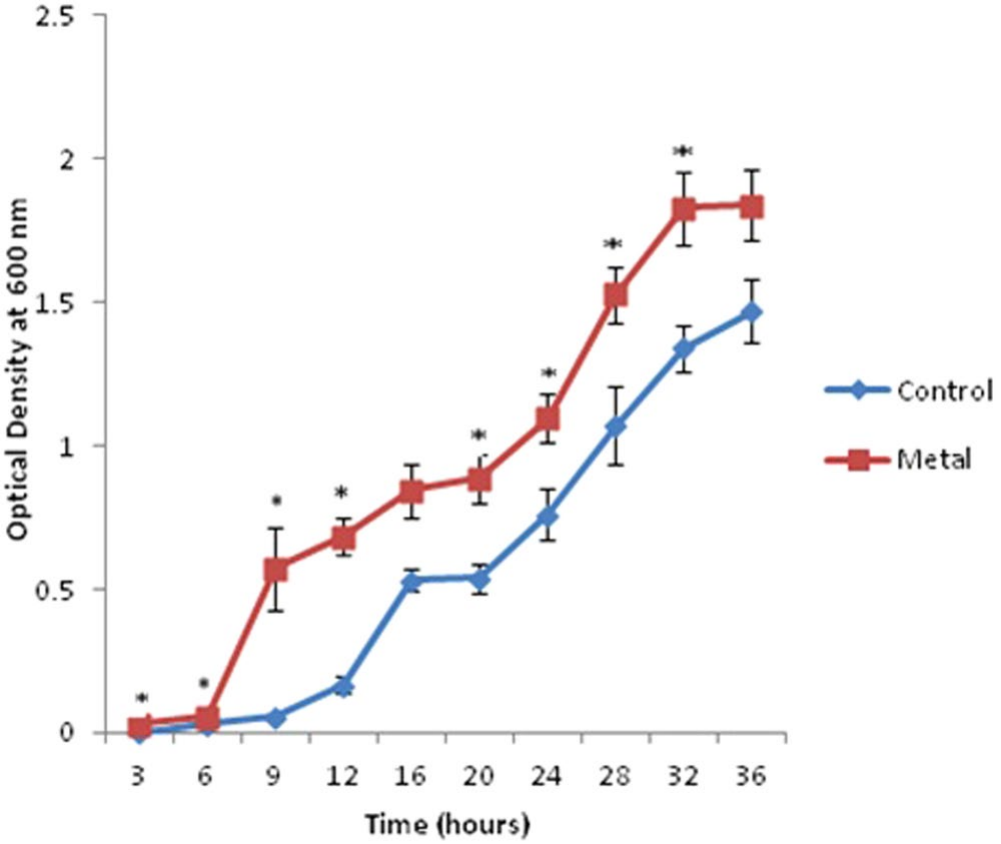
Growth curve of Halomonas sp. strain *BVR* 1 in the presence and absence of metal. The * represents significant difference (p ≤ 0.05) between control and metal treated sample within the same time period. Blue indicates control cells; Red indicates treated cells.

The trend of bacterial growth was similar between metal treated and untreated bacterial cells, as is evident from Fig. 1, though the metal treated cells exhibited significantly higher O.D values at all time periods. This observation is as per the expected outcome, as *Halomonas sp*. strain *BVR* 1^22^ has been isolated from a heavy metal rich effluent and is thus likely to be better adapted to heavy metals, thereby leading to enhanced induction of bacterial growth in the presence of metal^22^. For the first 6 h after sub culturing, the growth trend was identical in both control and metal treated bacteria. A sharp surge in growth was observed in the metal treated cells against the untreated ones. This indicates a shorter lag phase in the metal treated bacteria and could be a response due to the presence of heavy metal in the media. A less steep surge was seen in the control cells after 12 h of growth. Overall, similar growth rates were observed in both metal treated and untreated bacteria respectively.

### Analysis of Lead uptake by Bacteria

**Figure 2:**
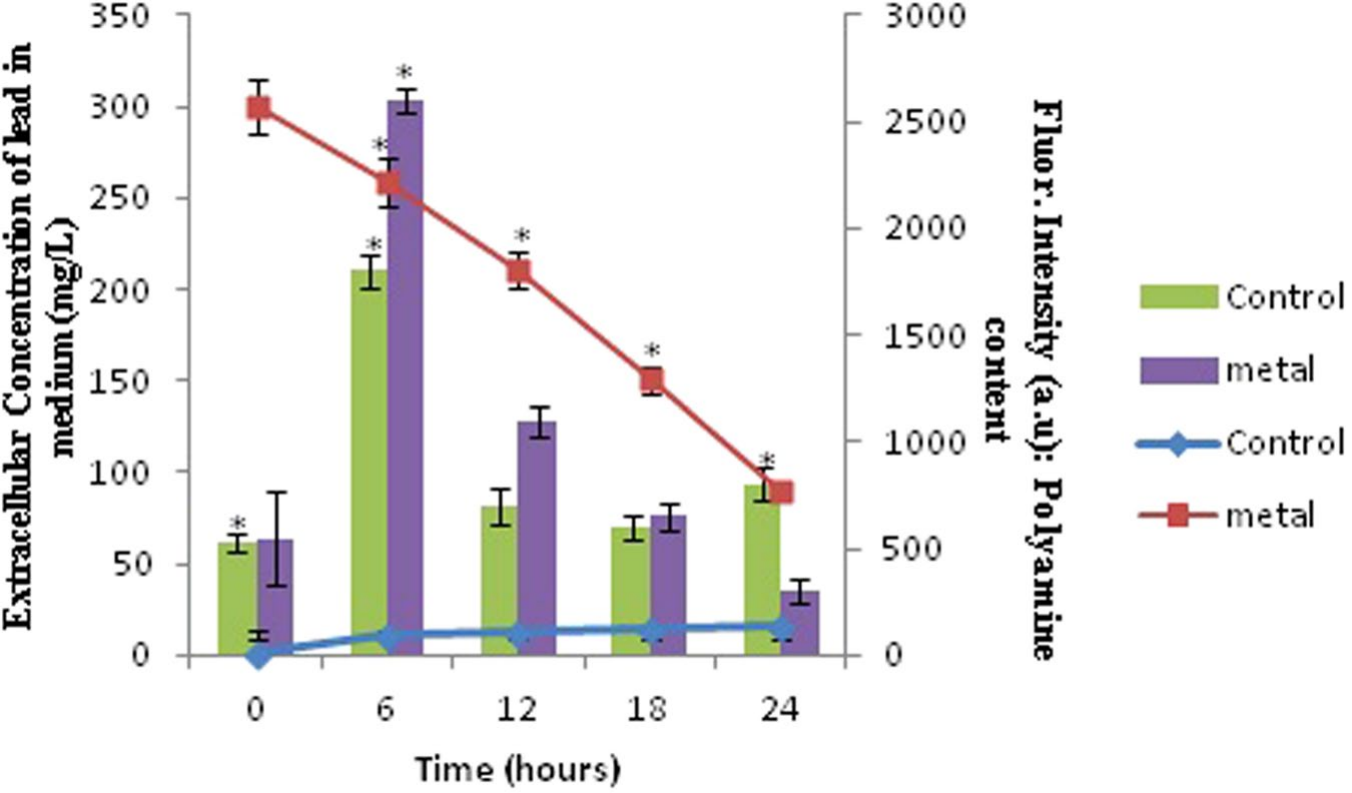
Comparing extracellular concentration of metal in growth medium and total polyamine content across various time points. Data are mean (±) standard error of 6 replicates from 2 experiments. The * represents significant difference (p ≤ 0.05) between control and metal treated sample within the same time period. Bars represent the polyamine data while line represents the extracellular concentration of lead in medium.

To establish that the Pb supplemented in the medium is indeed being endogenously taken up by the bacteria, we analyzed the disappearance of Pb in the spent medium using Atomic Absorption Spectrophotometry (AAS) (Fig. 2). In the treated, there was a gradual decline in the extracellular concentration of metal from a period of 6 h to 24 h (Fig. 2), indicating a relative uptake of metal ions by the cells. Overall, this decrease of extracellular metal concentration in the medium during the period of 6 h to 24 h in metal treated cells is an indirect measure of metal contributing to polyamine increase in these cells. The other mechanisms adopted by the bacteria to tolerate metal contamination include cell wall absorption, chelation, extracellular sequestration, etc^23^.

### Analysis of total Polyamines using Fluorescence Spectrophotometry

Polyamines are considered to be part of the General Adaptation Syndrome (GAS) generated in response to various environmental stresses, including heavy metal stress^11,24^. Heavy metals are directly involved in the redox reactions in cells and results in the formation of O_2_^·−^. This reactive oxygen species leads to the generation of H_2_O_2_ and ^•^OH and brings about the membrane disruption^25^. Polyamines are known to scavenge these Reactive Oxygen Species (ROS) and other free radicals thereby acting as antioxidants and helping in combating heavy metal stress^26^. *In-vivo* and *in-vitro* studies have suggested that in this way, they can effectively protect and stabilize the membrane systems against hazardous effects of redox active metal ions in bacteria^27^.

**Figure 3:**
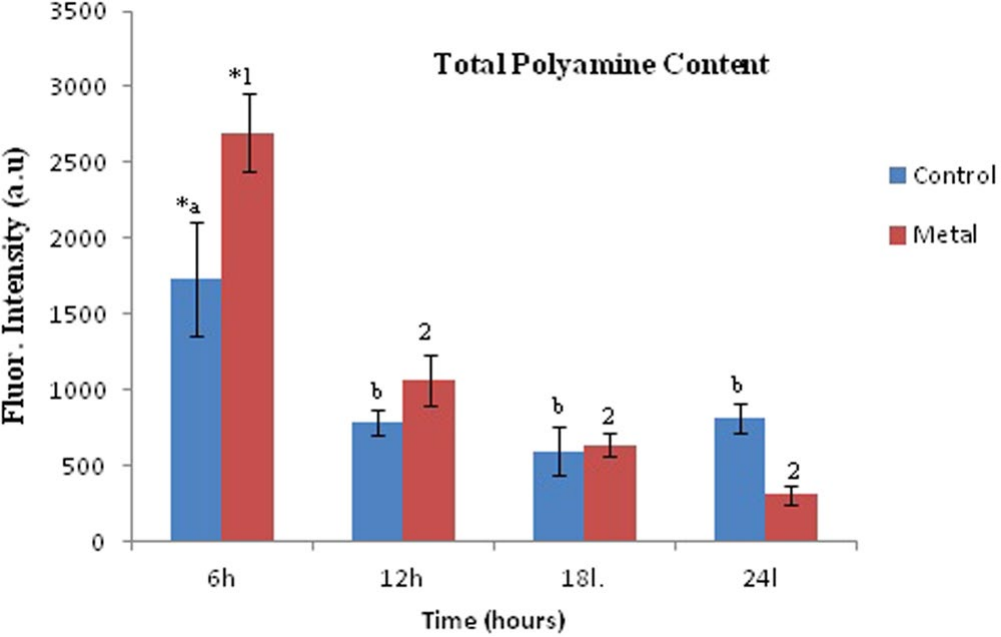
Total polyamine concentration in the control and metal treated cells as determined through fluorescence. Data are mean (±) standard error of 6 replicates from 2 experiments. *Represents significant difference (p ≤ 0.05)between control and metal treated samples within the same time period, a,b represents significant difference (p ≤ 0.05) between control samples across time periods and 1,2 significant difference (p ≤ 0.05) between treated samples across time periods. Blue indicates control cells; Red indicates treated cells.

Under normal conditions, endogenous levels of polyamines in cells are regulated by both, polyamine synthesis and catabolism. Simultaneous regulated expression of polyamine biosynthetic and degradation genes (like amine oxidases causing oxidative deamination of polyamines) is required to maintain the intracellular levels of polyamines^28^. As dansylated polyamines exhibit fluorescence^29^, the total polyamine concentration in the metal treated and control samples were calculated by the amount of fluorescence generated by these molecules when excited at 365 nm (emission was at 510 nm) (Fig. 3). Both control and metal treated samples exhibited an overall decrease in total PA concentration between 6 and 24 h post sub-culturing and metal addition.

Two way ANOVA test for both treated and control cells, depicted that the polyamine content at 6 h was significantly different from the results obtained across the other time periods. Individual trend analysis of control samples showed that, there was a significant decrease (about 2-folds) in fluorescence from 6 to 12 h, followed by insignificant fluctuations up to 24 h (decrease at 18 h followed by marginal increase at 24 h). In case of metal treated cells, a similar pattern with even more sharp and significant decrease (about 2.5-folds) was observed between 6 and 12 h post metal addition. This decrease continued subsequently all the way up to 24 h but turned out to be statistically insignificant. (as determined by 2-way ANOVA).

Comparison at individual time points indicated that the total PA concentration in metal treated cells was significantly higher at 6 h post metal treatment as compared to controls. The leveling out of the total PA concentration in the control samples post 12 hrs and a decreasing trend in the metal treated cells resulted in overall low PA presence in the metal treated cells as compared to controls in the 24 h time point.

The exact mechanistic role of polyamines in bacteria under metal stress is not well established. But based on literature available in plants, PAs are hypothesized to chelate the heavy metal ions^30^ reducing their availability for causing metal toxicity.

### Analysis of individual PAs using Mass Spectrometry

After understanding the trends in total PA concentration within the bacterial cell, it was important to assess and understand the trends with respect to the three individual PAs – putrescine, spermidine, spermine.

Since these are highly charged cationic molecules with a very small size, we used a well- established derivatization technique to tag the individual polyamines (details in materials and methods) and used LC-MS for their detection and quantification. Derivatization leads to a significant increase in the molecular masses of these polyamines depending on the number of the dansyl groups being attached. Hence, the resultant mass of the PAs does not impede with the masses of any other biological molecules. Spectral analysis was done in the positive mode, owing to a stronger signal in this mode as compared to the negative one. The electrospray mass spectra selected from the first quadruple gave a single charged protonated molecule at m/z 555 corresponding to putrescine. Spermine and spermidine are tetra and tridansylated polyamines respectively and gave their ion product spectra at m/z 1135 and m/z 845 respectively^31^.

**Figure 4:**
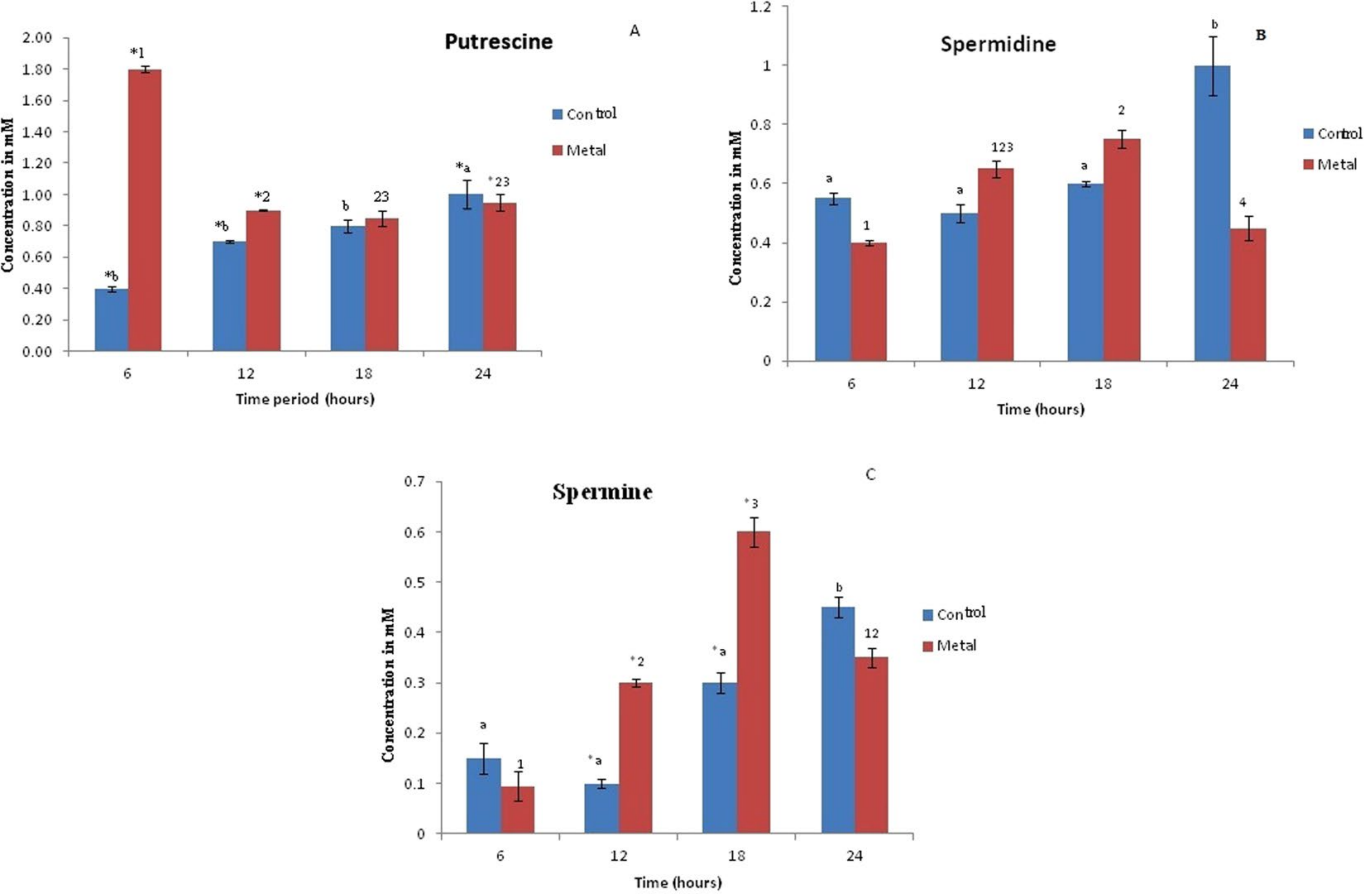
(**A**) Variation in putrescine levels (Obtained from mass spectrometry data) over a period of time. *Represents significant difference (p ≤ 0. 05)between control and metal treated samples within the same time period, a,b represents significant difference (p ≤ 0.05) between control samples across time periods and 1,2,3 represents significant difference (p ≤ 0.05) between treated samples across time periods. (**B**) Mass data of the variation in spermidine levels over a period of time. a,b represents significant difference (p ≤ 0. 05) between control samples across time periods and 1,2,3,4 represents significant difference (p ≤ 0.05) between treated samples across time periods. (**C**) Mass data of the variation in spermine levels over a period of time. *Represents significant difference (p ≤ 0.05) between control and metal treated samples within the same time period, a,b represents significant difference (p ≤ 0.05) between control samples across time periods and 1,2,3 represents significant difference (p ≤ 0.05) between treated samples across time periods. Data are mean (±) standard error of 6 replicates from 2 experiments. Blue indicates control cells; Red indicates treated cells.

The putrescine concentration gradually increased from 6 to 24 h in the control cells (the putrescine levels at 24 h was significantly higher from all the other time periods, as determined by 2 way ANOVA), an overall significant decrease in the same was observed in the metal treated cells (Fig. 4A).

Considering changes at individual time periods, at 6 h, the putrescine concentration in metal treated cells was significantly higher (about 5 folds) than the control cells. Significant differences in putrescine levels continued to exist in the metal treated vs. untreated cells at 12 h & 24 h period also, albeit to a lower extent. Overall, the cellular putrescine concentration increased significantly in the first 6 h of inducing heavy metal stress, correlating positively with bacterial growth at 6 h (Fig. 1). Earlier reports with plants have indicated similar enhancement in putrescine production as an early protective response against heavy metals, since putrescine is the precursor molecule for the production of other polyamines^7^. Putrescine levels increased significantly in apple callus under salt stress^32^. Metal stress (Cu^+2^) increased the putrescine levels in 7 day old raddish seedlings, aiding in oxidative stress management^38^.

The cellular concentration of spermidine in the treated cells shows a gradual increase from 6 h to 18 h following which the spermidine concentration decreases significantly at 24 h (Fig. 4B). On the other hand, there was a different pattern seen in untreated cells. The levels in the untreated cells did not show any change from 6 to 12 h followed by a significant increase at 24 h (as confirmed by the 2 way-ANOVA). Surprisingly (and negatively correlating with putrescine data), comparison at different time points indicated low spermidine in the metal treated cells as opposed to control cells at 6 h and 24 h. However, at 12 and 18 h the spermidine concentration was higher in treated cells as compared to control. None of these differences at individual time periods between metal and control cells were statistically significant though. Spermine cellular levels followed a similar trend like spermidine in both metal treated and untreated cells till 18 h (Fig. 4C). Spermine levels gradually increased in concentration over a time period from 6 h-18 h in case of metal treated cells. There was however a significant fall observed in the concentration beyond 18 h. In case of untreated cells, there was a dip in 12 h similar to spermidine and then a subsequent gradual rise in the levels beyond 12 h. The spermine levels at 24 h was significantly higher in comparison to all the other time periods, as showed by 2 way-ANOVA.

To summarize, in metal treated cells, over a period of time from 6 h to 24 h, putrescine concentration gradually decreased (Fig. 4A) with a concomitant increase in spermidine and spermine levels up to 18 h. Hence, it can be speculated that the synthesized putrescine is now being utilized for the synthesis of other polyamines (along with simultaneous, possible catabolism of putrescine) (Fig. 4A,B,C).

It is likely that, after an initial spurt of putrescine at 6 h, the other polyamines, spermine and spermidine start contributing towards defending the cell from the heavy metal. In our study, spermine increase was more predominant as compared to spermidine between the 6 h to 24 h period. This could be ascribed to the fact that the antioxidant activities of these polyamines are associated with the number of amine groups and spermine, being a tetra-polyamine is likely to be more efficient in scavenging ROS^34,35^. The levels increased significantly according to the bacterial cell density during the logarithmic and stationary phases. Production of all the polyamines shows a decline between 18 to 24 h indicating enhanced polyamine catabolism.

### FT-IR assay

**Figure 5:**
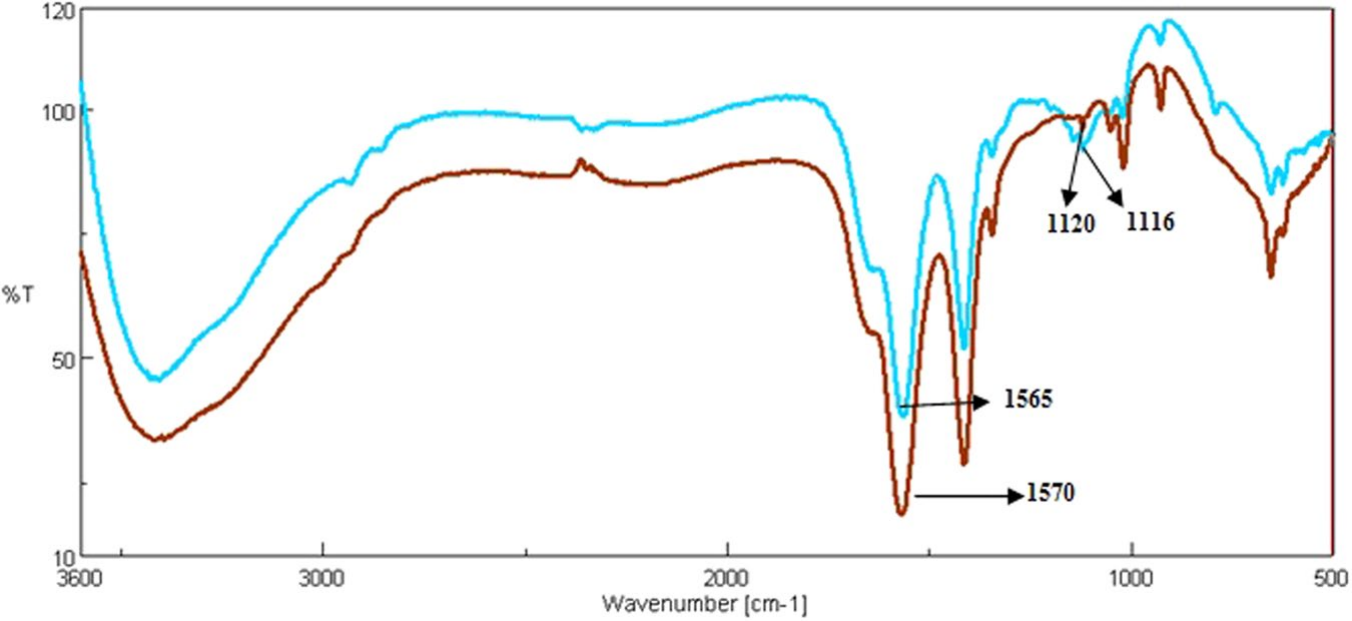
FTIR analysis of the polyamines from control and metal treated bacterial cells. (Red:Polyamines from metal treated samples, Blue: Polyamines from control samples).

FT-IR of the polyamines of the control and the cells exposed to the metal was carried out to determine whether the metals are involved in binding to the polyamines. Few characteristic changes have been observed in the FTIR spectrum of both control and metal treated samples. A shift in the peak was observed from 1565 cm^−1^ in the control samples to 1570 cm^−1^ in metal treated samples^36^. This wave number corresponds to the N-H vibrations. This shift in the peak was accompanied by a reduction in the percentage transmittance from 38.1943 to 18.2595. (Fig. 5) The C-N peak in the control sample may correspond to single C–N bonds that is present at 1116 cm^−1^ ^37^. This peak also shifted slightly to 1120 cm^−1^ in the metal treated samples. The results obtained from the FTIR analysis do suggest a role of these polyamines in direct metal chelation in these bacteria under high Pb conditions (Fig. 5).

### Cytotoxicity assay

**Figure 6:**
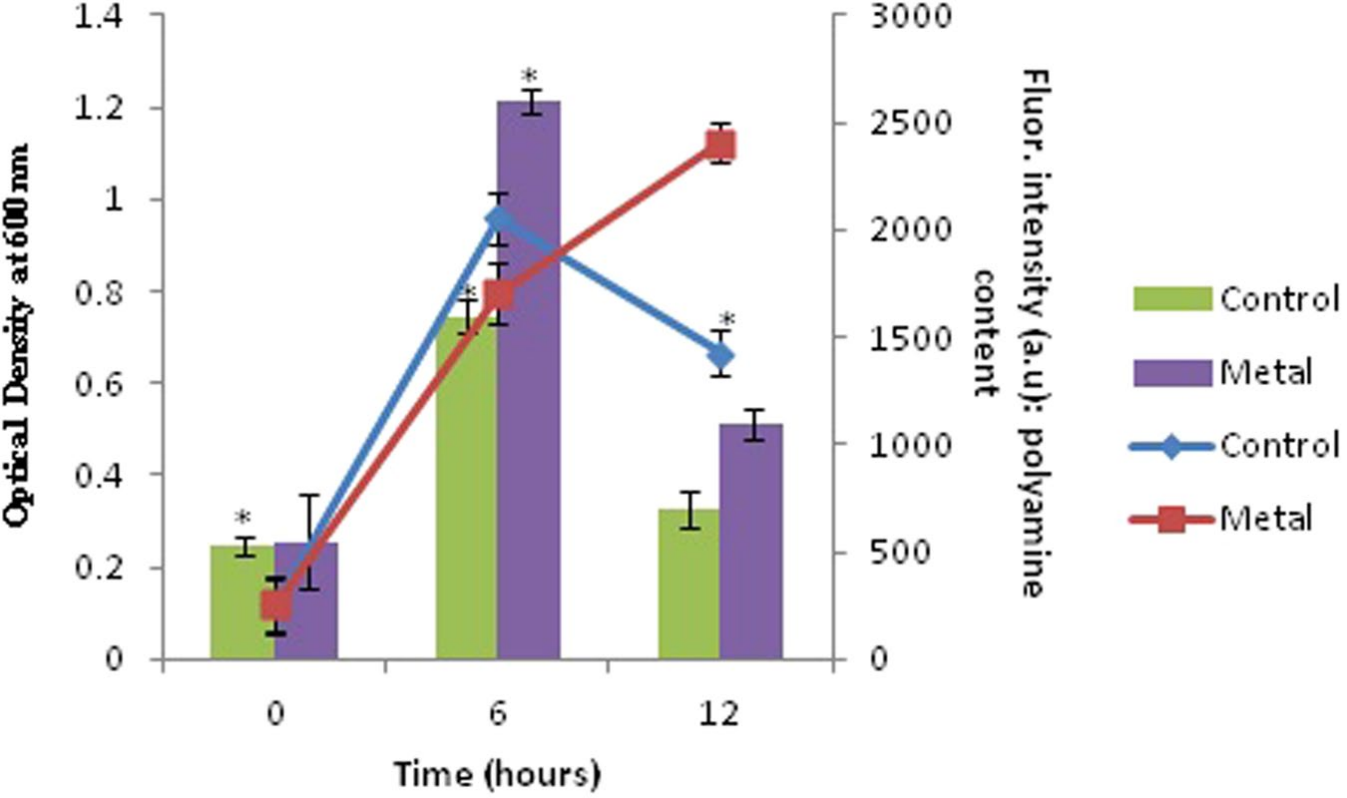
Comparing membrane damage assay of the cells in the presence of metal and total polyamine content at various time points. Data are mean (±) standard error of 6 replicates from 2 experiments. The * represents significant difference (p ≤ 0.05) between control and metal treated sample within the same time period. Blue indicates control cells; Red indicates treated cells. Bars represent polyamine content while line represents Optical Density at 600 nm.

Cell viability assays were done at 6 h and 12 h post metal addition. At a 6 h time period, when the cells are in lag phase, adjusting to the growth environment, there is no significant change in growth statistics in the control and metal treated samples as is evident from the Trypan blue and MTT assay. Results from Trypan blue assay can be very well supported with the growth curve data, where there is a surge in the growth at 6 h time period, indicating that there is not much membrane damage at this time interval and the cells are actively growing. At 12 h, where cells are in an actively dividing phase, the quantity of cells exhibiting membrane damage in metal treated cells was higher as compared to the untreated cells (Fig. 6).

**Figure 7:**
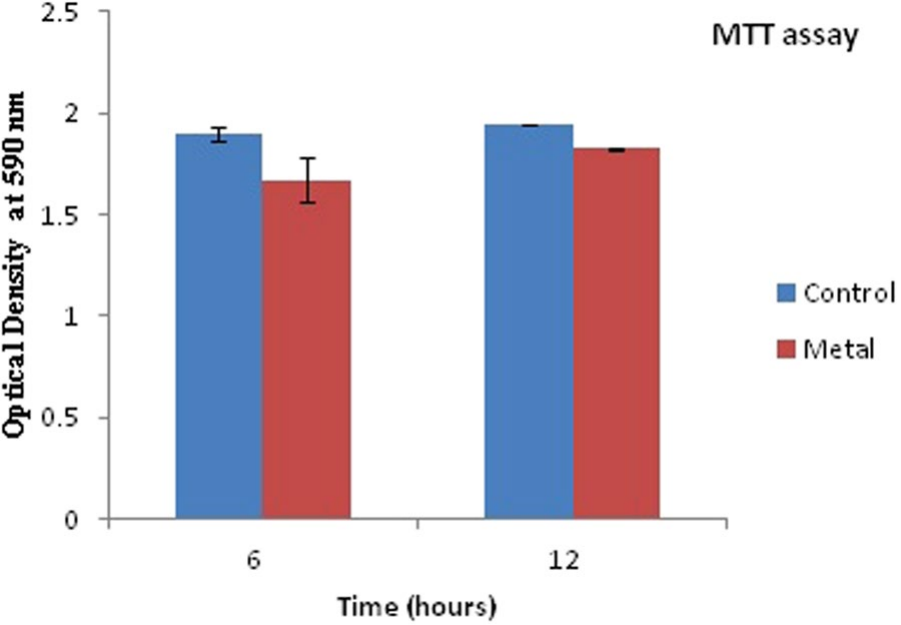
Respiratory activity of the cells in the presence of the metal. Blue indicates control cells; Red indicates treated cells.

No significant difference is seen in the MTT data between the control and metal treated cells, although it can be seen that membrane compromised metal treated cells show slightly low respiratory activity at 12 h as against the bacterial cells at 6 h post metal treatment (Fig. 7). In order to corroborate our data with the physiological status of *Halomonas sp*. strain *BVR* 1. Under Pb treated conditions, growth and viability assays were conducted to assess the physiological response of these bacteria to Pb treatment. These results point towards a complex regulation of physiological processes in these bacteria due to Pb addition. For example, at 6 h post metal treatment, the growth curve (Fig. 1) indicates a surge in bacterial growth, while the MTT assay (Fig. 7) does not exhibit a corresponding increase in respiratory activity of these bacteria. However, after a prolonged period (12 h) of post metal addition, the Trypan blue assay (Fig. 6) did exhibit an increase in membrane damage. In this context, further experimentation will be required to elucidate the reasons for some of these observations.

## Materials and Methods

Analytical grade reagents were used in the experiments.

### Bacterial growth and Metal stress

Heavy metal resistant bacterial strain, *Halomonas sp*. strain *BVR* 1 (KC178681) was isolated from an electronic industry effluent and has been identified after a detailed biochemical and molecular characterization involving 16S r- DNA sequencing^22^. This isolate was found to be resistant to a range of heavy metals and antibiotics. The Minimum Inhibitory Concentration (MIC) of the strain towards cadmium and lead was found to be 200 mg/L and 400 mg/L respectively, while it could tolerate zinc up to 250 mg/L and chromium up to 150 mg/L. For the analysis of modulations in PA levels in the presence of metal, the strain (being a halophile), was cultured overnight under aerobic conditions at 37 °C and constant shaking at 120 rpm in Luria Bertani broth (LB) supplemented with 3% NaCl and lead with pH of the medium set to 7.2 for better growth^38^. Growth medium supplemented with 300 mg/L of Pb^2+^ was inoculated with 100 µl of overnight *Halomonas sp*. strain *BVR* 1 (O.D. _600_ value of 0.2; approx. 1 × 10^4^ cells) to study the impact of heavy metal toxicity. This concentration of the metal solution was chosen to induce metal stress, as this was slightly lower than the calculated Minimum Inhibitory Concentration (MIC)^22^. Metal concentration in the medium was tested using a HI98185 ion meter equipped with lead ion selective electrode (Hanna Instruments, USA). There was no reduction in the metal concentration indicating no complexation with the constituents in the medium. Bacterial cells grown without lead supplementation were used as controls. Control and treated cells were grown and harvested at varied time intervals of 6 h, 12 h, 18 h and 24 h for further analysis.

### Analysis of Lead uptake by Bacteria

Analysis of lead uptake by bacteria was done by measuring Pb^2+^ concentration in spent Vs. fresh medium. About 25 mL of LB medium supplemented with 300 mg/L Pb^2+^ (treated cells) was inoculated with 100 µl of the overnight bacterial culture (O.D_600_ of 0.8; approx. 2 × 10^6^ cells) and incubated at 37 °C. Both the treated and control tubes were taken out at various time intervals of 6 h to 24 h. The samples were centrifuged at 5670 × g for 10 min. Subsequently, the supernatant was used for measurement of extracellular heavy metal concentration using Atomic Absorption Spectrophotometer (AAS).

### Extraction and dansylation of free, endogenous PAs

Polyamines were extracted by the standard freeze-thaw method as proposed by Minocha *et al*.^29^. Bacterial cultures were pelleted down, supernatant was discarded and about 450 ± 20 mg of cells was mixed with four volumes of 5% perchloric acid and frozen at −20 °C. Following three rounds of freezing and thawing, these samples were vortexed and centrifuged for 10–15 minutes. About 100 µl of the supernatant was collected and used for dansylation. To the supernatants, 100 µl of saturated Na_2_CO_3_ solution and 100 µl of dansyl chloride (in acetone) was added. After 1 h of incubation at 60 °C, 50 µl of 20 mg/mL asparagine made in distilled water was added to the above mixture. After an additional incubation of half an hour, about 400 µl of toluene was added. Samples were vortexed and allowed to stand for 5 min. These were then centrifuged at 18000 × g for 1 min to facilitate separation of aqueous and organic phases. Organic phase containing the polyamines was transferred to a new eppendorf tube and vacuum evaporated using a rotary evaporator. Samples were reconstituted using 1 mL of methanol.

Dansylation was also performed on PA standards in a similar fashion. The standards used were putrescine, spermidine and spermine (Sigma-Aldrich). Concentration of the standards ranged from 0.1 mMol L^−1^ to 1 mMol L^−1^, which falls in the same range as the physiological polyamine levels in an active bacterial cell^39^. An internal standard 1,7 diaminoheptane was used to account for error due to spillage, evaporation etc.

### Analysis of total PAs using Fluorescence Spectrophotometry

Total dansyl-PAs were analyzed using fluorescence spectrophotometry (Spectramax M4). The cell free extracts containing dansyl-PAs were analyzed at an excitation wavelength of 365 nm and an emission wavelength of 510 nm respectively against a blank sample as a reference^40^.

### Analysis of individual PAs using Liquid Chromatography Mass Spectrometry (LC-MS)

Liquid Chromatography Mass Spectrometry (LC-MS) was performed using a Shimadzu HP series to quantify the individual polyamines^31^. A C18 Column (Phenomenox) was used for the separation of PAs. The mobile phase used for the separation of compounds was a gradient established between acetonitrile (A) and water (B) both acidified with 0.l % formic acid. The gradient program was set up as follows: the gradient used was 0 to 2 min, 60% A/40% B. This was followed by a linear increase of B, reaching 100% at 8 min; from 8 to 10 min, 100% B; at 11 min, 40% A and 60% B; and from 11 to 15 min, 60% A and 40% B. The injected amount was 50 µl and the flow rate was maintained at 200 µl/min. The mass analysis was performed on a Shimadzu HP Series Single Quadrupole. The source was operated in both positive and negative mode at an ion spray voltage of 1000 V. The oven temperature was set to 25 °C at a flow rate of 1 mL/min. All these experiments were carried out in triplicates.

### Fourier Transform Infrared Spectroscopy (FT-IR) analysis

FTIR spectra was generated for polyamines extracted from the 6 h control and metal treated samples using a Jasco 4200 FT-IR spectrometer in the range 400–4000 cm^−1^. The samples were dried overnight, followed by encapsulation into dry KBr powder. The prepared pellet was then scanned and the spectra of polyamines from control and metal treated bacterial cells were recorded.

### Cytotoxicity assay

Cytotoxicity assays of the bacterial cells were carried out at 6 and 12 h post metal treatment using two methods - Trypan Blue method and MTT assay. The Trypan Blue assay is based on the principle that a larger quantity of the blue dye enters cells which have damaged/compromised cell membranes as opposed to cells with healthy/ intact membranes. Hence, injured cells tend to stain a deeper blue than healthier cells. For Trypan Blue assay about 100 mg wet weight of the bacterial cells at 6 h and 12 h time period were incubated in 1 mL of 0.05% Trypan blue dye for 15 minutes. This mixture was centrifuged at about 22000 × g for 15 min. The supernatant was discarded and the pellet was washed till the supernatant appeared colourless. The pellet was now resuspended in 1 mL of 1% SDS and was spun at 22,000 × g. Absorbance of the supernatant was measured spectrophotometrically (Beckman Coulter DU 730) at 600 nm.

The chemical 3-(4,5-dimethylthiazol-2-yl)-2,5-diphenyl tetrazolium bromide, abbreviated as MTT is used to test for cell viability by measuring respiratory activity. Colorless MTT interacts with the electron transport chain and is reduced to a blue colored product called formazan^41^. Thus, actively respiring cells tend to exhibit greater intensity of the blue color. The assay was carried out according to a standard protocol^42^. According to this protocol, 100 mg wet weight of the bacterial cell pellet harvested after 6 h and 12 h of growth was taken and suspended in 250 µg of MTT reagent, followed by gentle mixing at room temperature for an hour. The mixture was centrifuged at 22000 × g for 10 minutes. The supernatant was discarded and the pellet was resuspended in 1 mL of 0.04 Mol L^−1^ acid propanol. This suspension was again centrifuged to obtain the supernatant for further analysis. This supernatant was measured spectrophotometrically at 590 nm.

### Analysis of Growth curve

For growth curve measurement, about 20 mL culture volume of the LB medium, supplemented with 100 mg/L Pb^2+^ (permissible lead concentration for growth) was inoculated with 100 µl of the overnight bacterial culture (Optical Density at 600 nm (O.D_600_ of 0.8; approx. 2 × 10^6^)) and incubated at 37 °C. Bacterial growth was measured at 600 nm at regular time intervals of 6, 12, 18, 24, 30 and 36 h. Growth curve comparison of *Halomonas sp*. strain *BVR* 1 in the presence and absence of metal was analyzed.

### Statistical analysis

Statistical analysis was carried out using student’s t-test (Microsoft- Excel) or two factor ANOVA (Graph pad prism 7.0) as required.

## Conclusions

There is no clear information till date on the role of polyamines as a possible player in combating heavy metal stress in bacteria. This study adds to the existing literature of polyamines in bacteria and throws light on their heavy metal stress mitigation phenomenon. The results demonstrated in this paper, depicts a simple and easy measurement of polyamines using mass detection and fluorescence spectrophotometer in bacterial cells. A detailed analysis of the production of polyamines in bacterial cell under the exposure of heavy metal at varied time intervals has been studied.

It was evident that there was a significant increase in the levels of polyamines under metal stress particularly, putrescine, the precursor to the other polyamines (Spermidine and Spermine) in the initial 6 h. This was the major polyamine produced in the presence of heavy metal. However, a time dependent increase in the levels of spermine and spermidine was also observed in the bacterial cells till a period of 18 h, followed by a gradual dip in the production beyond this period of time was seen owing to its utilization in the stability of the cell and as a part of the cells protective response. The cytotoxicity assays shows that with increased exposure to heavy metal, there is a gradual increase in the membrane damage, as was observed at the 12 h time period.

The multitude of functions, which polyamines have, along with their connections through the complex metabolic cycle to many other metabolites, makes it difficult to delineate the clear role of polyamines in bacteria under metal stress. Also, the molecular mechanism’s involving polyamines and various heavy metal scavenging needs to be addressed.

## References

[1] Sharma SS, Dietz KJ (2006). The significance of amino acid and amino acid- derived molecules in plant responses. J Exp Bot.

[2] Moat, A. G, Foster, J. W. & Spector, M. P. Microbial stress responses. Microbial physiology. (Fourth Edition, Chapter 18) 582–600 (John Wiley & Sons, Inc. 2000).

[3] Ramakrishna A, Ravishankar GA (2011). Influence of abiotic stress signals on secondary metabolites in plants. Plant Signal Behav.

[4] Ron, E. Z. The prokaryotes-prokaryotic physiology and biochemistry, *Bacterial stress responses*. 589–598 (Springer, 2013).

[5] Rani, A. & Goel, R. Microbial strategy for crop improvement, 85–104 (Springer-Verlag Berlin Heidelberg, 2009).

[6] Chen L, Han Y, Jiang H, Korpelainen H, Li C (2011). Nitrogen nutrient status induces sexual differences in responses to cadmium in *Populusyunnanensis*. J Exp Bot.

[7] Pang XM, Zhang ZY, Wen XP, Ban Y, Moriguchi T (2007). Polyamines, all purpose players in response to environmental stresses in plants. Plant Stress.

[8] Paschalidis KA, Roubelakis-Angelakis KA (2005). Spatial and temporal distribution of polyamine levels and polyamine anabolism in different organs/tissues of the tobacco plant. correlations with age, cell division/expansion, and differentiation. Plant Physiol.

[9] Mohapatra S, Minocha R, Long S, Minocha SC (2010). Transgenic manipulation of a single polyamine in poplar cells affects the accumulation of all amino acids. Amino Acids.

[10] Gill SS, Tuteja N (2010). Polyamines and abiotic stress tolerance in plants. Plant Signal Behav.

[11] Hossain MA, Piyatida P, Jaime A (2012). Teixeira, da Silva. & Fujita, M. Molecular mechanism of heavy metal toxicity and tolerance in plants: Central role of glutathione in detoxification of reactive oxygen species and methyl glyoxal and in heavy metal chelation. Journal of Botany Article ID.

[12] Studham E, Macintosh GC (2013). Multiple phytohormone signals control the transcriptional response to soybean aphid infestation in susceptible and resistant soybean plants. Mol Plant Microbe In.

[13] Alcazar R, Altabella T, Marco F, Bortolotti C, Reymond M, Koncz C, Carrasco P, Tiburcio AF (2010). Polyamines: molecules with regulatory functions in plant abiotic stress tolerance. Planta.

[14] Tahri J, Sayel N (2014). H., Bahafid, W. & Ghachtouli, N. E. Effect of polyamines on the reduction of hexavalent chromium by bacterial strains and their resistance. Biotechnol Agron Soc Environ.

[15] Rhee HJ, Kim EJ, Lee JK (2007). Physiological polyamines: simple primordial stress molecules. J Cell Mol Med.

[16] Cozatl DM, Loza-Tavera H, Hernandez-Navarro A, Moreno-Sanchez R (2005). Sulfur assimilation and glutathione metabolism under cadmium stress in yeast, protists and plants. FEMS Microbiol Rev.

[17] Groppa MD, Tomaro ML, Benavides MP (2007). Polyamines and heavy metal stress: The antioxidant behavior of spermine in cadmium- and copper-treated wheat leaves. Biometals.

[18] Tan WG, Fu B, Li SF (2015). Metallomics and NMR-based metabolomics of chlorella sp. reveal the synergistic role of copper and cadmium in multi-metal toxicity and oxidative stress. Metallomics.

[19] Girdhar M, Sharma NR, Rehman H, Kumar A, Mohan A (2014). Comparative assessment for hyperaccumulatory and phytoremediation capability of three wild weeds. 3 Biotech.

[20] Casale A, De Stefano. C, Manfredi G, Milea D, Sammartano S (2009). Sequestration of alkyltin (IV) compounds in aqueous solution: Formation, stability, and empirical relationships for the binding of dimethyltin (IV) cation by N- and O-donor ligands. Bioinorganic chemistry and applications.

[21] Cicatelli A (2010). Arbuscular mycorrhizal fungi restore normal growth in a white poplar clone grown on heavy metal-contaminated soil, and this is associated with upregulation of foliar metallothionein and polyamine biosynthetic gene expression. Ann. bot.

[22] Manasi RV, Kumar ASK, Rajesh N (2014). Biosorption of cadmium using a novel bacterium isolated from an electronic industry effluent. Chem Eng J.

[23] Rajendran P, Muthukrishnan J, Gunasekaran P (2003). Microbes in heavy metal remediation. Indian J Exp Biol.

[24] Leshem Y, Kuiper PJC (1996). Is there a GAS (general adaptation syndrome) response to various types of environmental stress?. Biol Plantarum.

[25] Anjum NA (2014). Glutathione and proline can coordinately make plants withstand the joint attack of metal(loid) and salinity stresses. Front Plant Sci.

[26] Ha HC (1998). The natural polyamine spermine functions directly as a free radical scavenger. Proc Natl Acad Sci.

[27] Pegg E, Robert AC (2011). Current status of the polyamine research field. Methods Mol Biol.

[28] Rouached H, Pal S, Rachmilevitch S, Libault M, Lam-Son PH (2015). Plants Coping Abiotic and Biotic Stresses: A Tale of Diligent Management. Biomed Research International.; Article ID.

[29] Minocha SC, Minocha R, Robie CA (1990). High-performance liquid chromatographic method for the determination of dansyl- polyamines. J Chromatogr.

[30] Norris V, Reush RN, Igarashi K, Root-Bernstein R (2014). Molecular complementarity between simple, universal molecules and ions limited phenotype space in the precursors of cells. Biol Direct.

[31] Ducros V, Ruffieux D, Belva-Besnet H, Florence de Fraipont BF, Favier A (2009). Determination of dansylated polyamines in red blood cells by liquid chromatography-tandem mass spectrometry. Anal Biochem.

[32] Liu JH (2006). Polyamine biosynthesis of apple callus under salt stress: importance of the arginine decarboxylase pathway in stress response. J Exp Bot.

[33] Choudhary, S. P. C., Kanwar, M., Bhardwaj, R., Yu, J. Q. & Tran, L. S. P. Chromium stress mitigation by polyamine -brassinosteroid application involves phytohormonal and physiological strategies in Raphanus sativus L. *Plos One* **7**, 10.1371/journal.pone.0033210 (2012). 10.1371/journal.pone.0033210 PMC3315560 22479371

[34] Drolet G, Dumbroff EB, Legge RL, Thompson JE (1986). Radical scavenging properties of polyamines. Phytochemistry.

[35] Lovaas E (1997). Antioxidative and metal-chelating effects of polyamines. Adv. pharmacol..

[36] Ouameur AA, Tajmir-Riahi HA (2004). Structural analysis of DNA interactions with biogenic polyamines and cobalt (III)- hexamine studied by Fourier transform infrared and capillary electrophoresis. Biol Chem.

[37] Mejia VC (2014). Branched polyamines functionalized with proposed reaction pathways based on 1H-NMR, Atomic Absorption and IR Spectroscopies. Am J Anal Chem.

[38] Manasi RV, Kumar ASK, Rajesh N (2014). Adsorption isotherms, kinetics and thermodynamic studies towards understanding the interaction between a microbe immobilized polysaccharide matrix and lead. Chem Eng J.

[39] Shah P, Swiatlo E (2008). A. Multifaceted role for polyamines in bacterial pathogens. Mol Microbiol.

[40] Smith MA, Davies PJ (1985). Separation and quantitation of polyamines in plant tissue by high performance liquid chromatography of their dansyl derivatives. Plant Physiol.

[41] Mosmann T (1983). Rapid colorimetric assay for cellular growth and survival: application to proliferation and cytotoxicity assays. J Immunol Methods.

[42] Ikewage H, Yamamoto Y, Matsumoto H (1998). Cell death caused by a combination of aluminum and iron in cultured tobacco cells. Physiol Plant.

